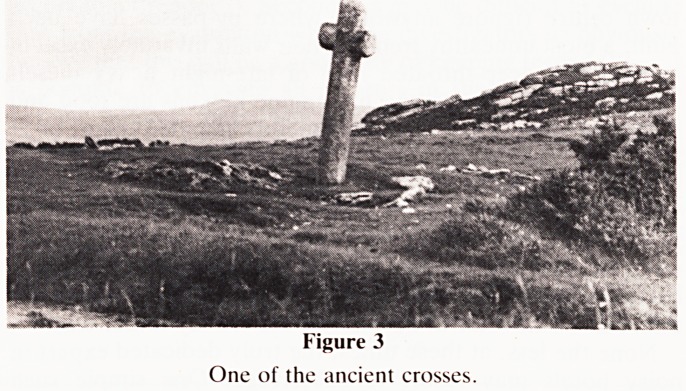# Dartmoor: A Place to Explore

**Published:** 1990-06

**Authors:** R. C. L. Feneley


### Dartmoor: A Place to Explore

**R. C. L. Feneley, MChir., FRCS**
Presidential Address, Bristol Medico-Chirurgical Society, October, 1989.

Rising majestically from the heart of the South-West peninsula, Dartmoor has been described as the highest, wildest land in England south of the Pennines. This area contains the richest concentration of archaelogical remains in the British Isles stretching over 365 square miles of remarkably contrasting countryside which was designated a National Park in 1951. The many relics of its history and the mysteries that they evoke still stand as if to challenge those who wish to search for them scattered and hidden away in this seemingly desolate moorland. This is not a place to wander far afield without being suitably clothed, booted and equipped with map, compass, whistle and light rations.

The unique feature of this part of Britain is the mountain blanket bog; this produces the notorious quaking mires such as the one at Fox Tor. Few visitors ever find the path which crosses that. The rainfall is heavy, often averaging 60 inches per annum or more, and the sodden peatland feeds the great rivers and their tributaries that arise on the moor such as the Okement and Taw to the North, the Teign and Dart to the East, the Erme and Yealm to the South and the Plym and Tavy to the West to mention but a few. The rivers have provided power for the local industries and a domestic water supply to Plymouth since 1591 when Drake constructed the leat named after him.

Benjamin Donn's map of Devon in 1765 showed no evidence of any archaeological features on the moor and in fact little was known about the moor until two acts of Parliament were passed in 1772 to authorise the building of a turnpiked road between Moretonhampstead and Tavistock. The development of the roads brought educated observers to the scene and evidence started to accumulate of man's long association with the moor. Leaf-shaped flints discovered alongside sites at Postbridge and Gidleigh Common and the chambered burial barrows at Coringdon Ball and Cuckoo Ball on the Southern moor probably date back over 5000 years to neolithic times. The Beaker Folk certainly appear to have visited the moor; these itinerant craftsmen travelled Europe extensively during the Bronze age from around 2000 BC to 500 BC. They introduced primitive metallurgy to the British Isles because their weapons and tools were made of bronze which is an alloy of copper with 10% tin. Bronze gave a sharper and longer lasting cutting edge to their tools and weapons which were constructed typically with a hole to take a shaft. These Folk have been recognised by the particular shape of beaker found in their graves. At Watern Oke in the upper reaches of the Tavy, a fine bell-shaped beaker was found in a Kistvaen or burial chamber and at Fernworthy a long-necked beaker was discovered together with a flint knife and a copper knife with a wooden hilt. The acid soil conditions of the moor unfortunately leave no remains of skeletons in the burial mounds and are harmful to metal objects left within them.

The stone circles, the stone rows and the standing stones or Menhirs have also been ascribed to the Beaker Folk, yet they form one of the most intriguing mysteries of the moor. Some stone circles surround a burial mound, forming a retaining circle whilst others are of considerable size, over 100 feet in diameter and are considered to have been used in religious ceremonies perhaps as temples. The stone rows may extend for very long distances; one on the western side of the river Erme measures over 11,000 feet and it ends in a stone circle that surrounds a burial mound at its southern extremity. Some stone rows are arranged in double or triple lines and are thought to have been used for processional purposes. During the past 1000 years numerous attempts have been made to exploit the moor. Tin, wool, peat, granite, naphtha, china clay and even gunpowder have all been produced at certain periods on or around the moor. The first documented evidence of tin near Sheepstor and Brisworthy appeared in the Pipe Roll of 1156. Tin mining developed on the moor in three phases; first there was the tin streaming in early medieval times, followed by the open cutting and adit mining during Tudor and Elizabethan times and finally the shaft mining of the 18th and 19th centuries when copper, iron, arsenic and lead were also discovered.

Tin was found on the moor in the form of its dioxide, Casserite, lying in thin veins or lodes in the granite. A fast flow of water was required to wash out the tin from the stones and in some places the rivers were narrowed to obtain this. Sandy Hole Pass is an excellent example of this on the East Dart which can be reached about 2½ miles above Postbridge. Smelting of the tin originally took place in two operations. First the rough drawing of the metal took place in a peat fire alongside the river but more careful refining then followed at a recognised smelting centre, probably at one of the stannary towns, namely Chagford, Ashburton, Tavistock or Plympton. About 300 tons of tin were produced per annum towards the end of the 12th century. The refining process improved vastly with the introduction of the blowing houses in the 13th century and these reduced the smelting to one procedure. The blowing houses were usually situated alongside a river; charcoal was used to fuel the furnace and a water wheel worked the bellows to stir the fire. The molten metal would be collected in a stone trough before transfer to a mould. The best preserved ruins of a blowing house can be found alongside the river Walkham above Merrivale Bridge. The Civil War appeared to stop mining exploits until the 19th century when a Bermondsey factory started to produce tin canisters for preserving food in 1812. This rapidly stimulated a revival of tin mining and introduced shaft mining with the discovery of other minerals on Dartmoor.

The wool trade owed its success to the monks, the Cistercians who settled at Buckfast in the 12th Century and the Benedictines at Tavistock. The tucking mills, again water-operated, where the wool was washed, treated and woven into cloth, started to develop in the towns around Dartmoor at Ashburton, Tavistock, Widecombe, Sticklepath and Belstone. Foreign trade arose with Florentine merchants and the scarlet cloth for the Nizam of Hyderabad's bodyguard was made at the Belstone mill.

The fascination of Dartmoor is intimately associated with the social and industrial history that is available to the visitor. Perhaps the best way to reveal this is to take the reader on a tour by road to visit a few of Dartmoor's antiquities. The route is not a difficult one but it takes the motorist across the moor towards Cornwall, a detour indeed but one that is well worth considering if time permits. An Ordnance Survey map and a compass are not essential for this trip but they greatly enhance the interest by helping to identify the landmarks, particularly the Tors and the Hills silhoueted on the skyline.

The journey starts at Bovey Tracey (Fig. 1), taking the road up the hill to Hay Tor, past the Headquarters of the Dartmoor National Park which is well worth a visit as so much information is housed there. Do make sure of taking the left fork for Hay Tor rather than the right one which goes to Becky Falls and Manaton. On reaching the top of the hill, the moor suddenly opens up and the broad causeway leading up to Hay Tor can be seen ahead. At this point the road lies very close to the old granite tramway (Fig. 2) which was built by George Templar in 1820 to carry granite from the Haytor and Holwell quarries to Teigngrace and thence by water to

**Figure 1**

The journey starts at Bovey Tracey.

London. Templar had anticipated the building of London Bridge which started in 1825, but Dartmoor granite was also used for many other buildings in London including New Goldsmiths' Hall. Look out for a turning to the right for Manaton and stop the car at any convenient place. The tramway can be found a few yards onto the moor from the right side of the road and followed up to the right side of the Hay Tor peaks. The granite point system is worth a visit but let the imagination run free for a moment to imagine the flat-topped wagons with their load of granite running down hill with only a primitive pole applied to the rims of the wheels to act as a break. The tramway closed in 1858 as the railways began to take over transportation. After that brief stop, drive on up the hill, past Saddle Tor on the right and Rippon Tor on the left, bearing to the right past Hemsworthy Gate. Take the left turn for Widecombe in the Moor and pass down Northway. There is a convenient stopping place on the left hand side of the hill, ideal for a picnic, with a panoramic view across the valley. At the bottom of the hill lies the small church of Saint Pancras, a rare dedication, built in the late fourteenth century of perpendicular style. The high tower with its large crocketed pinnacles was added in the late 15th or early 16th century. To the right of the picture, the huge mass of Hameldon dominates the scene and further to the North the attractive Chinkwell and Honeybag Tors can be seen.

**Figure 2**

The old granite tramway.

Moving on to Widecombe, the Church is certainly worthy of a visit. According to the list of rectors and vicars there had been a church on this site before the present building and in 1260 Bishop Bronescombe of Exeter gave authority for the inhabitants of Pizwell and Babeny, ancient local tenements, to bury their dead at Widecombe rather than carry the bodies across the moor on the arduous journey along the Lich Way to Lydford.

The Cathedral-in-the-moor as it is often called, was built from the proceeds of tin and wool. Disaster struck the church on Sunday 21st October 1638, during the afternoon service when a violent thunderstorm broke over the valley and a thunderbolt penetrated the church roof, killing 4 worshippers and injuring 62. The local school master wrote an official account of the tragedy and this can be read in the church on a large panel at the base of the tower.

The legend that surrounds the tragedy typifies Dartmoor folklore, explaining the event as an act of the Devil. A local rascal, Jan Reynolds had previously sold his soul to the Devil and on that fateful day the Devil had come to collect him whilst he sat in the back of the church playing cards. Suspicion had been aroused in the neighbourhood that an unwanted visitor was in the vicinity. A stranger had visited a local inn and ordered a glass of ale from the barman. It was reported that there was an audible sizzling as he drank the ale and steam was noted to be issuing out of his nostrils. When the Devil called at the church, he tethered his coal-black steed to one of the pinnacles of the church, captured Jan from the congregation, climbed the Tower and threw him across his horse. As he did so, the horse lashed out with his hooves, breaking off the pinnacle of the church which crashed through the roof inflicting the horrific injuries on the congregation.

On leaving Widecombe head initially for Ponswothy but then strike north for the Moretonhampstead road passing along the west side of Hameldon to visit Grimspound, the early bronze age village which lies below Hameldon Tor. The pound was built with a massive encircling wall and it contains the remains of 24 hut circles. Each hut circle was built with a low stone wall about 12–15 feet in diameter and a single entrance facing South or South West. Rafters would be erected from the wall to form a cone at the top like a wigwam and the roof consisted of a covering of heather or rushes. Bronze age settlements were of two types grouped either in pounds such as Grimspound with a wall enclosing the village as a protection from marauders or the nucleated village with a long string of dwelling houses without an encircling wall. The nucleated village at Watern Oke on the edge of the Amicombe Brook above Tavy Cleeve is the largest settlement on Dartmoor. There are remains of about 1500 hut circles scattered over the moor.

On reaching the Moretonhampstead to Tavistock road, B3212, turn left and drive for about $1\frac{1}{2}$ miles, before stopping at the car park beside an ancient cross. This has been called Benet's Cross although there is controversy about the spelling and whether Benet was a tinner from Chagford or a monk from Buckfast Abbey. The letters W.B. are graven on to the shaft of the cross which stands at site of the old trans-Dartmoor track that was used in the Middle Ages but the cross became a bond stone to mark the Warren Bounds probably in the late 18th century. The car park is a good vantage point to view the local scene which was an area of intense activity at the Birch Tor and the Vitifer Mines with many of the ruins of the open adit mining still visible. The last tin to be extracted from the moor in fact came from the dumps of these two mines in 1939 when they were taken away for smelting.

The Warren House Inn lies ahead on the right hand side of the road where the traditional food would have been scrumpy and rabbit. Rabbits were introduced into Britain in the 12th century and there are remains of a number of warrens on the moor. There was one behind this Inn but the Trowlesworthy and Ditsworthy Warrens are more impressive. It is worth calling in for a drink as the fire has been burning in the hearth non-stop since the present Inn was opened in 1850. The original Inn was situated on the other side of the road and appears on Donn's map of Devon (1765) as the New Inn before being demolished in 1845.

Postbridge is the next compulsory stop to view the famous clapperbridge which was used by the packhorses to cross the East Dart river before the turnpike was built. The bridge measures 42 ft 8 in long and the surface consists of four immense slabs of granite, the largest of which is 15 ft 2 in. in length and 6 ft 9 in. wide. The central waterway is spanned by two slabs of granite and in 1874, one of these lay in the river after a local man had attempted to form a wall across the river to prevent the ducks from going too far downstream! It was replaced in 1890 but William Crossing, the famous Dartmoor guide, pointed out that it is now upside down and inside out.

Leaving Postbridge with Archerton Farm on the right side and Bellever Forest on the left, look out for Powder Mill Farm on the right after about 2 miles. George Frean established a rock powder factory there in 1844. Rock powder was produced by a mixture of 15 parts of charcoal, 75 parts of saltpetre and 10 parts of sulphur. The powder was ground in a trough on a circular stone head which was turned by a water wheel supplied from the East Dart by leats. The remains of the buildings can still be visited; they had thick walls but the roofs were very light in case an explosion occurred. Silas Sleep worked at the Powder Mill and his wife would pack up his breakfast and lunch for him before he left his home at Postbridge. The story relates how Silas would eat both his breakfast and lunch as soon as he arrived for work because he was afraid that he might be blown up before he had time to enjoy his lunch. The quality of the rock powder was tested by firing an iron ball from the mortar that now stands by the cottages. The next stop is at Two Bridges but look out for Crockern Tor on the right hand side just after passing Parson's Cottage. Crockern Tor was the seat of the ancient Stannary Parliament where the elders from the four Stannary Towns would hold court to consider the laws and rules of the tin trade. Offenders could be thrown into the prison at Lydford such as Richard Strode, the local Member of Parliament who tried to limit their activities. The last sitting of this court took place in the early 18th century.

From Two Bridges Wistmans Wood is an interesting place to visit. It is one of the three ancient dwarfed oak woods on Dartmoor but a walk of about $1\frac{1}{2}$ miles is required heading north past the farm towards Crow Tor. The trees are stunted, distorted, pedunculated oaks covered with lichens and mosses and estimated to be about 500 years old. The other two woods, namely Piles Copse on the river Erme and Black Tor Beare are more remotely situated and less impressive.

Princetown owes its identity to Sir Thomas Tyrwhit who was an undergraduate at Christ Church Oxford with the Prince of Wales. Probably as a result of their acquaintance, he became the Prince's Secretary in 1786 and he built Tor Royal which is a large establishment at the Prince's Town as it was known. He was to become Lord of the Stannaries in 1805 and he was knighted in 1812. He was responsible for building the prison for the French prisoners of war who were confined in hulks on the rivers in and around Plymouth. The foundation of the prison was laid in 1806 and the building was sufficiently advanced in 1809 for 2500 men to be housed there after marching from Plymouth. The war ended, the prisoners left in 1816 and the buildings lay vacant until 1850 when the prison was reopened for convicts. When visiting Princetown, it is worth taking the road past Tor Royal to the White Works which stand in an isolated position facing the infamous Fox Tor quaking mire. With a pair of binoculars, it is possible to make out the site of Childe's Memorial Tomb on the moor below Fox Tor. The story of Childe the Hunter is a perfect example of folklore based on fact. Childe was caught in a snowstorm on the moor when his steed died, so he disemboweled the horse, climbed inside the carcass and wrote a note to the effect that whosoever gave his body a Christian burial could inherit his lands at Plymstock. He died in the storm and the monks of Tavistock found his body before the people of Plymstock could reach it. They took the body back to Tavistock to discover that a group from Plymstock were barring the bridge so they threw across another bridge a little higher up the Tavy which is called Gilde Bridge and thence buried the body and claimed their inheritance. According to Finberg in Devonshire Studies, Childe was a title of honour and there was a giant Devon landowner who is buried at Tavistock and who was a hunter.

The final part of the journey takes the road from Princetown to Tavistock passing the television mast on North Hessary Tor. At Rundlestone on a fine day there is a magnificant view to the West and within this area of the moor so much of interest to explore. It is worth stopping at the car-park on the left side of the descent and taking a careful look for the landmarks. Walk towards the television mast and there are standing stones marked DCP for Dartmoor Convict Prison that date back to the early 19th century. To the South the old quarries can be visited at Foggin Tor and the track of the original tramway built by Thomas Tyrwhit which carried the granite to Plymouth can be seen. Granite from this quarry was used for Nelson's Column in Trafalgar Square. To the West there are the Merrivale antiquities consisting of one single and two double stone rows each with a blocking-stone, a stone circle and a fine menhir that stands over ten feet high. To the North, the outcrops of Little Mis and Great Mis Tors can be seen and a walk to the latter Tor provides a panoramic view of the Northern Moor. If an O/S map and compass are at hand, it is possible to identify the Great Links Tor and Amicombe Hill to the North-West, High Willhays and Yes Tor to the North, Cocks Hill and the Great Kneeset to the North-East.

**Figure 3**

One of the ancient crosses.

Passing on down the hill to Merrivale, the bridge crosses the River Walkham and it is a little north of this point that the ruins of a blowing house may be found. Up the sharp hill on the other side, one passes the last remaining granite works on the moor with its quarry; this was a busy area for the granite workers and at the top of the hill, there is evidence of the open-air benches on the side of Little Steeple Tor. The granite sett-maker would probably have been active on these slopes around 1870 cutting the stone for the roads and pavements in Plymouth. In this area there are examples of "feather and tares" on some of the large boulders where pins have been hammered into them to split the granite.

A car-park is situated strategically on the left side of the road on Whitchurch Common before the descent from the moor into Tavistock. Take a final stop at this point to have a

*Continued Page 61*

*Dartmoor: A Place to Explore*
*Continued from page 59*

close look at one of the ancient crosses (Fig. 3) which is unique amongst those on Dartmoor in being chamfered. It is thought to date back to the 15th century and was probably used by the monks from Tavistock carrying their wool to direct them to the trans-Dartmoor track.

Darmoor may indeed appear bleak and desolate yet within the area of this National Park there is such abundance of history that a lifetime of exploration and interest is ensured. As a centre for recreation there are so many challenging walks, not least the pilgrimage to Crammere Pool where the concept of a small cairn to leave ones visiting card was started in 1854 by James Perrott, the famous Dartmoor guide who took Charles Dickens there. There are now over one hundred pillar boxes over the moor, hidden away at the strategic sites. Dartmoor was an ancient hunting ground and surely it remains a place for the adventurous to hunt but now for a different type of spoil.

### FURTHER READING

Dartmoor—A New Study, Edited by Crispin Gill, David and Charles, 1970.
High Dartmoor, Eric Hemery, Hale, 1983.
Worth's Dartmoor, Edited Spooner and Russell, David and Charles, 1967.
Crossing's Guide to Dartmoor, Brian Le Messurier, David and Charles, 1965.

## Figures and Tables

**Figure 1 f1:**
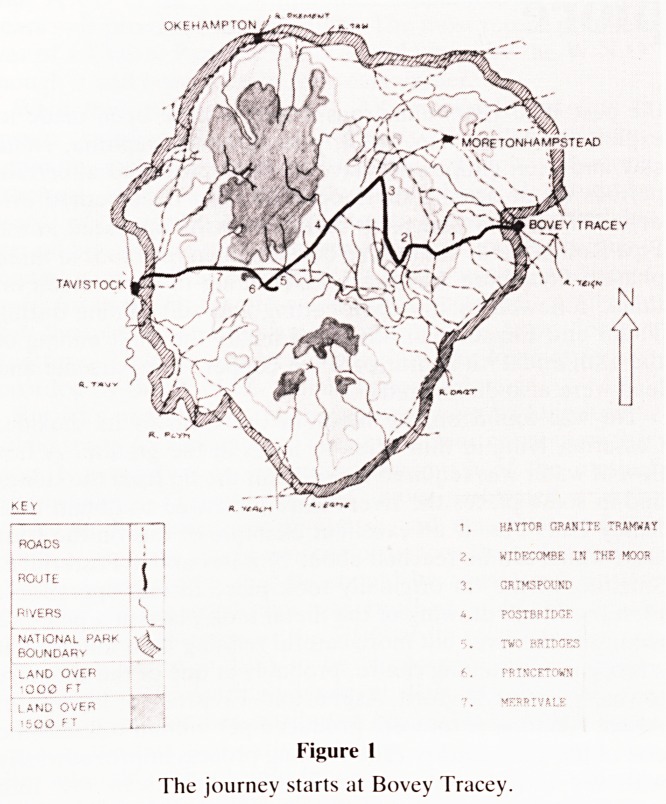


**Figure 2 f2:**
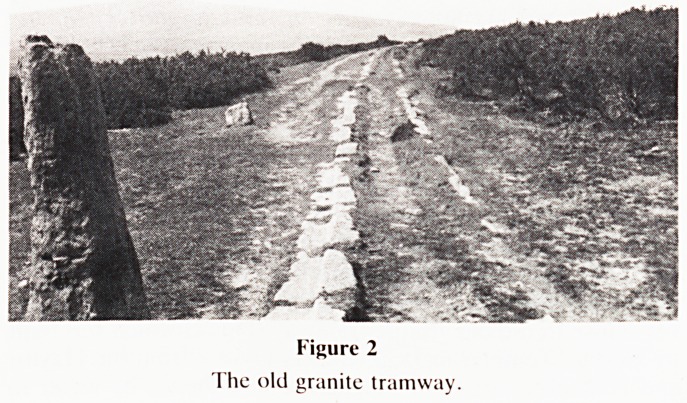


**Figure 3 f3:**